# Analysis of potential health impacts of road and rail traffic noise, using noise at residential locations in Austria as an example

**DOI:** 10.1007/s00508-025-02609-4

**Published:** 2025-09-05

**Authors:** Markus Hamik, Stefanie Schindler, Hanns Moshammer

**Affiliations:** 1 Center for Public Health, Department of Environmental Health, Medical University of Vienna, Kinderspitalgasse 1, 1090 Vienna, Austria; 2 AGES—Austrian Agency for Health and Food Safety, Institute for Medical Microbiology and Hygiene Vienna, Department for National Reference Centers, Währinger Straße 25a, 1090 Vienna, Austria

**Keywords:** Environmental exposure, Risk assessment, Environmental epidemiology, Community exposure, Non-communicable diseases

## Abstract

**Background:**

Environmental noise, particularly from road and railway traffic, has been identified as a significant public health concern. The World Health Organization (WHO) has highlighted the adverse effects of noise exposure on cardiovascular health, including ischemic heart disease (IHD). Despite the European Union’s regulations on air pollution, there are no mandatory limits for environmental noise exposure, necessitating further investigation into its health impacts.

**Methods:**

Noise exposure data were obtained from strategic noise maps and linked to Geographic Information System (GIS) data of Austrian buildings. Mortality data covering 5 years (1 Nov 2016 – 31 Oct 2021) were analyzed using Poisson regressions to evaluate the association between noise exposure at residential locations and mortality, specifically focusing on IHD. The analysis adjusted for age, sex, and noise bands, with sensitivity analyses to assess the robustness of the findings.

**Results:**

The study included 37,066,299 individuals, with 372,638 deaths recorded over 5 years. Higher noise bands were associated with increased incidence rate ratios (IRR) for IHD and all-cause mortality. The IRR for IHD increased by approximately 3% per 5 dB increase in noise levels. Sensitivity analyses confirmed the robustness of these findings, with stronger effects observed for railway traffic noise compared to road traffic noise.

**Conclusion:**

The findings underscore the significant health impacts of transportation noise, particularly on cardiovascular mortality. These results support the need for stricter noise regulations and comprehensive health impact assessments to mitigate the adverse effects of environmental noise exposure in Austria.

**Supplementary Information:**

The online version of this article (10.1007/s00508-025-02609-4) contains supplementary material, which is available to authorized users.

## Introduction

In the preparation of the 2018 Environmental Noise Guidelines (ENG) [1], the World Health Organization (WHO) Europe has mandated several systematic literature reviews and meta-analyses [2–4] regarding effects of environmental noise on cardiovascular and metabolic diseases, sleep disturbance, and annoyance.

While the European Union (EU) has set mandatory limit values for air pollutants that have been recently strengthened [5], limit values for environmental noise exposure are still missing. Instead, the EU has mandated member states to annually report the number of traffic noise exposed citizens per 5 dB zones. In 2019 [6], this regulation has been strengthened and reporting of the number of attributable cases, using the regression formula provided by WHO, is now also mandatory [1].

Highly annoyed and highly sleep disturbed persons are the largest group here [7] but much larger political and societal interest is with cardiovascular diseases, and its mortality. For example, regarding road traffic noise (ROTN), the WHO working group [1] rated findings on the incidence (from three cohort and four case-control studies) of ischemic heart disease (IHD) as high quality evidence. On the other hand, while in principle also supporting the findings on incidence, the cross-sectional studies on prevalence were rated as low quality. The three studies (one case-control and two cohort studies) on mortality included in a meta-analysis also found similar relative risks but the pooled estimate did not reach significance and the overall evidence for mortality was rated as moderate quality. Therefore, unsurprisingly, Annex III of the European Directive [6] mandated the reporting of the attributable incidence of IHD, assuming an 8% increase per every 10 dB increase in ROTN with a threshold of 53 dB (A-weighted L_DEN_).

Since the publication of the 2018 ENG, numerous newer studies have examined the effects of noise on various diseases and lots of research on the underlying mechanisms of noise-induced health effects has been made [8–10], including a broad meta-analysis [11], which also included recent studies that have specifically investigated the impact of noise on the incidence of IHD: among others a pooled analysis of Danish and Swedish cohorts [12] and a nationwide Danish study with over 2.5 million participants [13]. Münzel et al. (2024) concluded that a 10 dB(A) increase in ROTN was associated with a relative risk (RR) of 1.04 (95% confidence interval, CI, 1.02–1.06) for IHD incidence [11].

In order to demonstrate that the new reporting obligations imposed by the EU are based on scientific evidence, and that the overall quality of the data reported within the European Economic Area (EEA) [7] is reliable and applicable to the Austrian context, it seemed prudent to analyze the health effects of transport noise at residential locations using Austrian data. Given the relative ease of access, it was decided to utilize mortality data for this purpose.

## Methods

Noise exposure data were obtained from the open access strategic noise maps provided by the Austrian Federal Ministry for Climate Action, Environment, Energy, Mobility, Innovation and Technology [14]. Data are available from years before its update in 2022 for main roads, main railways, including major urban agglomerations each, and large airports; in 5 dB intervals for both nighttime noise (45–> 70 dB) and day-evening-night weighted noise levels (50–> 75 dB). There was a lack of data for Austrian airports in Geographic Information System (GIS) format, so the study was restricted to ROTN and railway traffic noise (RWTN).

The Austrian Federal Office of Metrology and Surveying offers public access to a Geographic Information System (GIS) database of Austrian buildings and their coordinates [15], which were used for further analysis. Buildings with sufficient data quality (approximately 80%) were included. Each property number is linked to a coordinate. If, however, this coordinate lies outside the actual building due to inaccuracies in data recording, a clear attribution to the traffic noise exposure of the respective residential property is not feasible. In such cases, the corresponding building and its inhabitants are excluded from the analysis. Buildings with a known non-residential use were excluded. The building’s GIS coordinates were compared to the geographical noise data and the noise level at the louder facade of each building. If it was located in two different noise bands, an aliquot number of persons was attributed to each. Noise levels were assessed for each source and each time (nighttime versus daytime weighted).

Aggregated data on persons (address, sex and 5‑year age group) were available from Statistics Austria. Building register data were linked to household data and that to each person. Data were obtained for 5 years (1 November 2016 to 31 October 2021). For persons at risk, every person registered in Austria at the beginning of each year at any building in the database was counted. From the address registry, information was available if the person was still registered at the same address at the end of the year, and if the person has already been registered in Austria for 10 years or more.

Next, person identification numbers were linked to the mortality register for the same year to evaluate if persons have died in the same year. Deaths from all causes as well as deaths from IHD (I20–I25 according to the International Classification of Diseases, 10th Revision [ICD-10], German Version 2013 [16]) were counted per noise band.

Thus, for further statistical analysis, the following data were available: per noise band for both ROTN and RWTN and for both daytime weighted and for nighttime noise the number of persons of each sex and each age group living in that noise band for the given years as a sum (persons living there for several years were counted multiple times) and the number of deaths (total deaths/deaths from IHD) by sex and age group. In a sensitivity analysis it could be discerned if a person was only registered at the beginning of the year at a given address (all persons), or if the person was also registered at the same address at the end of the year (remaining persons). Also, the analysis could be restricted to persons registered in Austria for at least 10 years (nationals).

Data were entered into STATA (Version 17; StataCorp, College Station, Texas, USA) and Poisson regressions were performed with number of cases as the outcome, the number of people at risk as the exposure variable and age-group, sex and noise-band as the independent variables.

The primary model used all non-exposed individuals as the reference population, whereby exposure always referred to the specific source under investigation, while other exposure sources could still be present. In an alternative model, individuals from the lowest exposure category were used as reference. In addition, separate analyses were run for death from all causes and from IHD and for “all persons”, for “remaining persons” and for “nationals”.

## Results

In total, 37,066,229 persons were covered by a valid address at the beginning of the 5 years (on average 7,413,245.8 per year), which represents a coverage of 84% of the Austrian population. Of these, 372,638 persons died over the 5 years (on average 74,527.6 per year). The vast majority of all persons lived in the reference zone, i.e., they were not exposed to any reported noise.

**Fig. 1:**
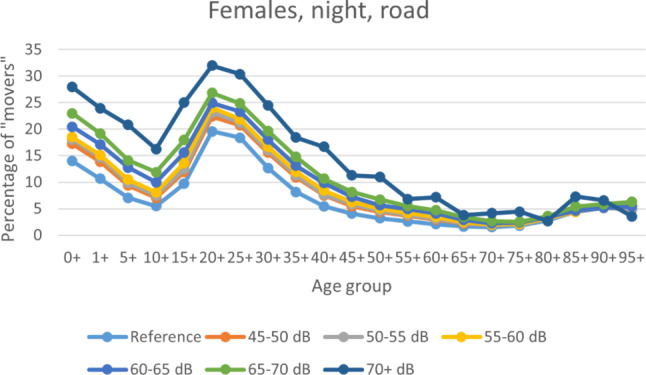
Percentage of “movers” ((all persons—remaining persons)/all persons in percent) demonstrated for females and night-time ROTN, per age group (in years)

**Fig. 2:**
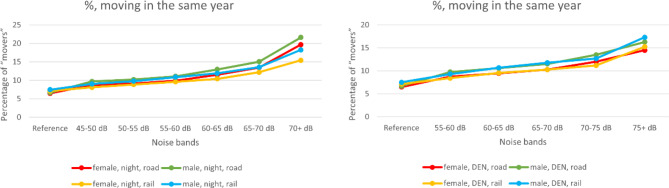
Percentage of “movers” ((all persons—remaining persons)/all persons in percent) for night-time (left) and 24‑h noise (right) for both sexes and for both ROTN and RWTN

Among the persons registered at any address at the beginning of each year, the percentage of those who changed that address before the end of the year (“movers”) was higher the higher the noise band. This was true for all exposure scenarios, for each sex and (nearly) all age groups (see Fig. 1 and 2). A pronounced peak in mobility was observed in the 20–30 year age group across all noise bands, likely reflecting typical patterns of young adults moving out. In contrast, mobility was low in older age groups, indicating greater residential stability among the old.

In all models examining mortality from all causes, males always displayed a higher risk than females with an incidence rate ratio (IRR) around 1.5, and the risk increased with age with an IRR around 1.7 per 5‑year age group and the lowest IRR in the age group 1 to under 5 years. For IHD, these findings were similar, and males displayed a higher risk than females with an IRR around 1.9, and the risk increased with age with an IRR around 1.9 per 5‑year age group and the lowest IRR in the age group 5–10 years.

**Fig. 3:**
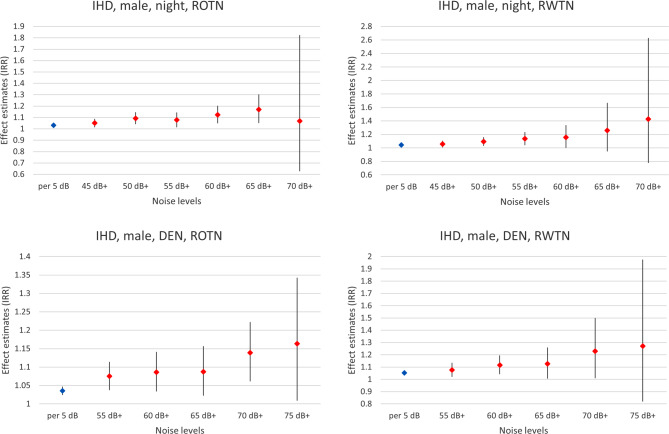
Effect estimates (IRR) per 5 dB increase in noise levels and/or compared to the non-exposed as the reference regarding deaths from IHD, for males, at night (upper panels) and DEN-weighted 24‑h noise levels (lower panels) for ROTN (left) and RWTN (right). Data are based on “remaining persons”

Higher day-evening-night weighted (DEN) noise bands were associated with higher IRR for IHD for both sexes and for all exposure scenarios. Apart from the highest noise band, the increase in the IRRs was fairly linear (see Fig. 3 for males (all persons) and Annex figures A1–A3 for females and for remaining persons).

**Fig. 4:**
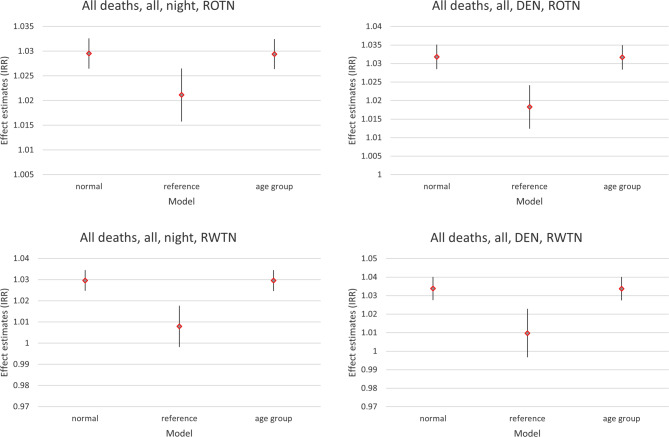
Effect estimates (IRR) per 5 dB increase in noise levels regarding deaths from all causes, for both sexes combined, at night (left) and DEN-weighted 24‑h noise levels (right panel) for ROTN (upper) and RWTN (lower panel). Comparison of 3 models: (1) “normal”: as in Fig. 3, for “remaining persons”, (2) “reference”: persons in the lowest noise band used as reference instead, (3) “age group”: first and second age group left out in order to compare “all persons” to “nationals” (Fig. 5)

Leaving out data from the youngest two age groups (in order to compare “all persons” to “nationals”, where the latter, per definition, must have been older than 10 years), did not alter the overall effect estimates. This is demonstrated in Fig. 4, which also compares the effect estimates based on the non-exposed as reference group with those based on those in the lowest noise band as the reference group.

**Fig. 5:**
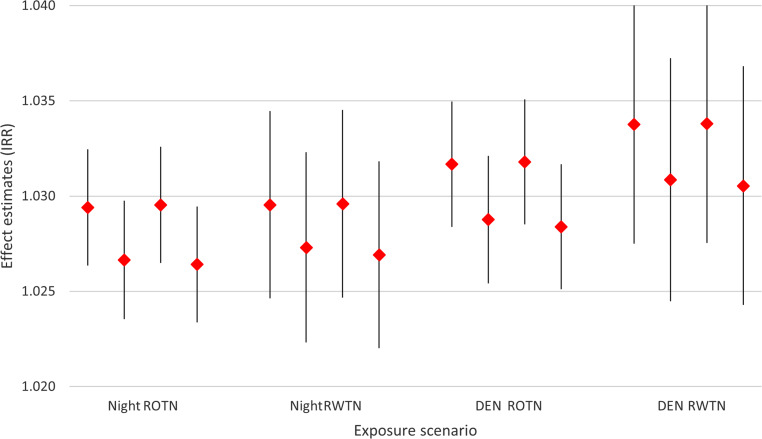
Effect estimates (IRR) per 5 dB increase in noise levels regarding deaths from all causes, for both sexes combined, for 4 exposure scenarios (nighttime and 24‑h noise, ROTN and RWTN). Comparisons between “all persons” and “remaining persons” (first and second sign from the left) and between “all persons (minus the first two age-groups)” and “nationals” (third and fourth sign from the left)

Figure 5 compares the effect estimates of the “remaining persons” to those of “all persons” and the “nationals” to those of “all persons” minus the youngest two age groups, with only small differences. Figure 5 also compares the exposure scenarios, where RWTN tends to display somewhat stronger effects than ROTN, and 24‑h weighted noise levels are slightly stronger than night-time noise levels alone.

The overall effect estimates for all kinds of noise exposures and regarding both deaths from all causes and from IHD are similar across sexes. Therefore, in the Annex tables, only effect estimates for both sexes are presented. The choice of the group at risk (all persons, remaining persons or nationals) and especially of the reference category does have an effect on the effect size though. This is shown in Figs. 4 and 5 as well as in Annex table A1. All effect estimates for different groups, models and outcomes are presented in the Annex (Annex tables A1–A6). Using the lowest exposure group as the reference category generally resulted in smaller effect estimates, with this reduction being more pronounced for RWTN. Thus, whereas using the non-exposed group as the reference category yielded stronger effect estimates per 5 dB increase in noise levels for RWTN than for ROTN, the opposite was observed when the lowest exposure group served as the reference, with smaller effects for RWTN compared to ROTN.

## Discussion

This study has in principle a register-based cross-sectional design. The advantage here is the coverage of a large number of individuals: more than 80% of the Austrian population over 5 years but even with this large cohort, case numbers, especially for deaths from IHD, became low in most age groups and especially in the higher exposure categories, causing imprecise effect estimates. Nevertheless, the most important shortcoming of the study design is the lack of individual information regarding possible confounders [17].

The most important possible confounder here is smoking history, which is clearly associated with higher IHD mortality. If smoking is also associated with noise exposure, it can act as confounder. An association between noise and smoking is very plausible, and two possible pathways must be considered: (A) noise exposure causes stress and could therefore either make it more likely to start smoking (especially if exposure begins earlier in life) or may increase smoking or hinder cessation. If noise exposure causally influences smoking behavior, smoking acts as a mediator, not a confounder, and adjusting for it would underestimate the true total noise effect. (B) People with lower socioeconomic status could not be able to afford more expensive housing in quiet areas and would also tend to smoke more or more likely. In that case, smoking could be seen as a confounder but it could also be argued that noise induces socioeconomic segregation. Noise, through either biological or psychological/behavioral or social/socioeconomic pathways would still cause the full total effect. Nevertheless, the lack of socioeconomic data on an individual or at least on the area level with sufficient spatial resolution, must be acknowledged as the most severe weakness of this register-based study. On the other hand, the effect of noise on health and especially on cardiovascular health is already well established. The current study did not set out to prove this association but rather to demonstrate the public health relevance of this established causal association in the context of a specific country for the whole population.

Instead of controlling individual confounders, sensitivity analyses were performed and two different reference populations were used: all non-exposed individuals and individuals from the lowest exposure category (see methods). While the second group (consisting of individuals residing near major roads or railways, but at a greater distance compared to their more exposed neighbors) was more comparable to the exposed population in terms of socioeconomic characteristics, the first group offered a larger sample size, which both have their own strengths and weaknesses. Indeed, when using the lowest exposure group as reference, the effect estimates were generally smaller; however, when looking at the IRRs of the lowest exposure category compared to the non-exposed group, this first ratio was always significant and strong, even though at least some exposed persons, e.g., from minor roads, might be spuriously put into the no-exposure group. Contrary to the expectation, this misclassification did not reduce the effect estimate, instead the ratios between consecutive noise bands decreased with increasing noise levels. For RWTN, effect estimates even declined in the highest exposure bands, though with wide confidence interval. The large ratio between the two reference groups suggests potential confounding, indicating that non-exposed individuals were not representative of the exposed but the declining ratios at higher noise levels might also reflect either an attenuation of effect, as seen in air pollution studies [18, 19], or exposure misclassification at higher noise levels.

There are two possible sources of exposure misclassification here: (A) houses situated in a noisy environment usually have been adapted to that environment through better insulating windows, locating bedrooms on quieter building sides and assigning exposed building fronts to non-housing purposes. The noise level at the facade at a specified altitude would be misleading in that case. (B) As this study has shown, with higher noise levels individuals are more likely to move away to another address and the cumulative exposure, which is likely more relevant for (chronic) diseases and mortality, will also be overestimated by the modelled noise levels. Both mechanisms could cause a reduced long-term exposure. This fact might even be more apparent for RWTN (where the drop in effect estimates at higher noise levels was often more pronounced), because most of the railway lines in Austria are relatively old and therefore, the building stock had ample time to adapt to that exposure. Only some new high-speed lines have been constructed in more recent decades but with great effort to minimize noise exposure in residential areas nearby.

Another possible source of bias is air pollution from the same source. This is relevant for ROTN, where noise and air pollution are likely highly correlated. The slightly stronger effect of ROTN compared to RWTN when using the lowest exposure group as the reference category may indicate such confounding; however, when the non-exposed group was used as the reference, effect estimates for RWTN were slightly stronger than for ROTN. This suggests that confounding by road traffic-related air pollution is unlikely to be a major factor.

Health effects of noise, especially on mortality, typically have a long latency period. Noise exposure during 2016–2021 is certainly not a perfect measure of lifelong exposure. There are three reasons for that: (a) some individuals have changed their residential address before. (b) New roads and railways have been built in the past; however, more recent projects have generally applied stricter noise mitigation (e.g. greater distance from settlements, noise barriers). Thus, high exposures according to the strategic noise maps often originate from older roads and railways. (c) The strategic noise maps do not cover all roads, although main roads are included. Therefore, this limitation primarily affects the lower exposure bands.

The extent to which these errors are differential is uncertain. Changing of address was more common among individuals in higher exposure bands, but movers were predominantly young adults. As mortality (for all causes and from IHD) mainly affects older age groups, exposure from middle age onwards is likely to be more relevant.

As in many epidemiological studies on environmental noise exposure, only residential noise exposure was examined. People usually are not confined to their home address throughout the day. Especially during daytime, they are often away for work, school, shopping or leisure. At night, they are more likely to remain at home, which may explain why night-time noise often shows stronger effects on health than 24‑h noise exposure metrics. Also, a main pathway to the health effects might be through repeated sleep disturbance by noise.

In our analysis, however, no clear difference emerged between nighttime noise and day-evening-night-weighted noise levels. This could indicate that residential noise exposure correlates with noise exposure at work: individuals living in poor and noisy environments may also tend to work in similarly noisy work settings. This finding again underscores the complex interconnections between socioeconomic status and noise exposure and raises questions of environmental justice.

In addition, the specificity of the observed effects warrants further consideration. There is substantial evidence that chronic noise exposure increases the risk of IHD [20], supported by plausible biological mechanisms [21]. Thus, it is reasonable to assume an increased IHD mortality, although epidemiological evidence is limited [22]. Mechanisms linking noise exposure to overall mortality are less clear, but some evidence suggests associations with deaths from other causes, particularly diabetes mellitus [23]. In the Austrian data set, over the 5 years, 58,852 deaths of all 372,638 deaths or 15.8% were due to IHD. Diagnostic misclassification remains a possible bias in register-based data.

Therefore, examining total deaths should be preferred over deaths from specific causes. Especially, when a cause of death is very frequent, as for IHD, the increased risk should also affect total deaths. Indeed, effect estimates do not differ substantially between these two (Annex figure A4). Besides a wider confidence interval for IHD cases, as expected, there might be some evidence for a stronger effect on the specific cause of death for RWTN, but not for ROTN. The cause for this, however, may also lie in the data source: the dataset only contains the primary cause of death, even if multiple causes are present. Therefore, among the non-IHD deaths, there could also be cases of IHD-related deaths, which would weaken the effect.

It was not possible to include individuals from the start to the end of the year, counting either the occurrence of death or loss to follow-up (change of address), as detailed information on residents at the start of the year and all internal migration movements were lacking. As address information was only available at the year’s beginning, it is possible that individuals who died had moved beforehand. Therefore, all persons, not just those remaining, should have been considered at risk. As moving frequency increased with noise levels (Figs. 1 and 2), focusing only on remaining individuals led to a stronger underestimation of persons at risk in higher exposure groups. Although death counts were unaffected, incidence rates were consequently overestimated. Nevertheless, as shown in Fig. 5, this bias was minor. It could also be argued that the remaining individuals represent a more stable cohort, potentially reducing bias.

Despite the advantages of a register-based cross-sectional study design, there are also some disadvantages in execution: lack of daily registration data, insufficient description of (potentially) available data for planning studies, strict requirements regarding sensitive personal data protection, and other bureaucratic hurdles. Furthermore, the lack of harmonization of the cut-off dates of different statistics and the unavailability of individual data are also detrimental to the planning and execution of studies like this.

Overall, the study found an increase in incidence rate ratios of around 3% per 5 dB increase in exposure. This would roughly translate into an increase of around 6% per 10 dB, which complies with the effect described by the WHO guidelines [1]. Additionally, as also noted in previous studies [11, 24, 25], the findings of our study underscore the importance of recognizing the impact of transportation noise on health and factor for the global burden of disease. This consideration is crucial on both a broader and an individual level, shaping policy decisions on noise limits, urban planning, and transportation strategies, while also playing a role in clinical assessments of risk factors to better evaluate individual patients’ risk for various diseases.

Because it seems unlikely that the EU will set binding limit values for environmental noise exposure soon, it is highly recommended that national member states, including Austria, set their own binding limit values based on WHO ENG recommendations.

## Supplementary Information

Annex
