# Supplementary material for: Analysis of potential health impacts of road and rail traffic noise, using noise at residential locations in Austria as an example
Source: Wien Klin Wochenschr. 2025 Sep 5;138(5-6):129–36. doi: 10.1007/s00508-025-02609-4 (PMC12992443; doi:10.1007/s00508-025-02609-4)
Supplement: Supplementary file 1 — Annex [file 508_2025_2609_MOESM1_ESM.docx]

Analysis of Potential Health Impacts of Road and Rail Traffic Noise, Using Noise at Residential Locations in Austria as an Example / Analyse möglicher Gesundheitsfolgen von Straßen- und Schienenverkehrslärm am Beispiel der Lärmexposition an Wohnstandorten in Österreich

# Supplementary Material

|  | IRR | 95% confidence interval | p-value |
| --- | --- | --- | --- |
| **All causes of death, remaining persons, both sexes, per 5 dB, compared to no exposure** |  |  |  |
| Night-time road | 1.02953 | 1.026482; 1.032589 | <0.001 |
| Night-time rail | 1.02959 | 1.024683; 1.03452 | <0.001 |
| 24-hour weighted road | 1.03179 | 1.028514; 1.035084 | <0.001 |
| 24-hour weighted rail | 1.0338 | 1.027537; 1.040102 | <0.001 |
| **All causes of death, remaining persons, both sexes, each noise band compared to no exposure** |  |  |  |
| Night-time road, 45-50 dB | 1.04362 | 1.032997; 1.054356 | <0.001 |
| Night-time road, 50-55 dB | 1.07764 | 1.06285; 1.092634 | <0.001 |
| Night-time road, 55-60 dB | 1.07813 | 1.059806; 1.096774 | <0.001 |
| Night-time road, 60-65 dB | 1.13221 | 1.110719; 1.154113 | <0.001 |
| Night-time road, 65-70 dB | 1.11022 | 1.075811; 1.145725 | <0.001 |
| Night-time road, 70+ dB | 1.24893 | 1.088571; 1.43292 | 0.002 |
| Night-time rail, 45-50 dB | 1.05866 | 1.044493; 1.07301 | <0.001 |
| Night-time rail, 50-55 dB | 1.06024 | 1.042538; 1.078233 | <0.001 |
| Night-time rail, 55-60 dB | 1.08862 | 1.061551; 1.116379 | <0.001 |
| Night-time rail, 60-65 dB | 1.11279 | 1.066609; 1.16097 | <0.001 |
| Night-time rail, 65-70 dB | 1.03208 | 0.9417933; 1.13102 | 0.499 |
| Night-time rail, 70+ dB | 0.98931 | 0.8005909; 1.22251 | 0.921 |
| 24-hour weighted road, 55-60 dB | 1.05769 | 1.046858; 1.068632 | <0.001 |
| 24-hour weighted road, 60-65 dB | 1.07119 | 1.0561; 1.086502 | <0.001 |
| 24-hour weighted road, 65-70 dB | 1.08829 | 1.069405; 1.107505 | <0.001 |
| 24-hour weighted road, 70-75 dB | 1.13506 | 1.112512; 1.158056 | <0.001 |
| 24-hour weighted road, 75+ dB | 1.11701 | 1.071693; 1.164242 | <0.001 |
| 24-hour weighted rail, 55-60 dB | 1.05999 | 1.043594; 1.076635 | <0.001 |
| 24-hour weighted rail, 60-65 dB | 1.06538 | 1.044198; 1.086994 | <0.001 |
| 24-hour weighted rail, 65-70 dB | 1.10473 | 1.069417; 1.141213 | <0.001 |
| 24-hour weighted rail, 70-75 dB | 1.11071 | 1.045407; 1.180087 | 0.001 |
| 24-hour weighted rail, 75+ dB | 0.98024 | 0.846821; 1.134684 | 0.789 |
| **All causes of death, remaining persons, both sexes, per 5 dB, compared to lowest noise band** |  |  |  |
| Night-time road | 1.02113 | 1.01576; 1.026527 | <0.001 |
| Night-time rail | 1.00787 | 0.9981155; 1.017713 | 0.114 |
| 24-hour weighted road | 1.01827 | 1.012387; 1.024189 | <0.001 |
| 24-hour weighted rail | 1.00970 | 0.9966729; 1.022899 | 0.145 |

Annex table A1: Effect estimates (IRR) for all causes of death, remaining persons, both sexes.

|  | IRR | 95% confidence interval | p-value |
| --- | --- | --- | --- |
| **All causes of death, all persons, both sexes, per 5 dB, compared to no exposure** |  |  |  |
| Night-time road | 1.026408 | 1.023368; 1.029458 | <0.001 |
| Night-time rail | 1.026911 | 1.022017; 1.031829 | <0.001 |
| 24-hour weighted road | 1.028393 | 1.025123; 1.031673 | <0.001 |
| 24-hour weighted rail | 1.030533 | 1.024289; 1.036815 | <0.001 |
| **All causes of death, all persons, both sexes, each noise band compared to no exposure** |  |  |  |
| Night-time road, 45-50 dB | 1.038075 | 1.027507; 1.048752 | <0.001 |
| Night-time road, 50-55 dB | 1.072089 | 1.057376; 1.087007 | <0.001 |
| Night-time road, 55-60 dB | 1.069594 | 1.051414; 1.088088 | <0.001 |
| Night-time road, 60-65 dB | 1.119569 | 1.09832; 1.14123 | <0.001 |
| Night-time road, 65-70 dB | 1.091172 | 1.057355; 1.126071 | <0.001 |
| Night-time road, 70+ dB | 1.205567 | 1.050772; 1.383166 | 0.008 |
| Night-time rail, 45-50 dB | 1.055197 | 1.041081; 1.069505 | <0.001 |
| Night-time rail, 50-55 dB | 1.054544 | 1.036942; 1.072445 | <0.001 |
| Night-time rail, 55-60 dB | 1.081131 | 1.054248; 1.1087 | <0.001 |
| Night-time rail, 60-65 dB | 1.102004 | 1.05627; 1.149717 | <0.001 |
| Night-time rail, 65-70 dB | 1.018574 | 0.9294691; 1.11622 | 0.694 |
| Night-time rail, 70+ dB | 0.9657 | 0.7814862; 1.193337 | 0.747 |
| 24-hour weighted road, 55-60 dB | 1.052432 | 1.041655; 1.063321 | <0.001 |
| 24-hour weighted road, 60-65 dB | 1.064863 | 1.049859; 1.080081 | <0.001 |
| 24-hour weighted road, 65-70 dB | 1.07856 | 1.059845; 1.097605 | <0.001 |
| 24-hour weighted road, 70-75 dB | 1.120791 | 1.098531; 1.143503 | <0.001 |
| 24-hour weighted road, 75+ dB | 1.095131 | 1.050702; 1.141439 | <0.001 |
| 24-hour weighted rail, 55-60 dB | 1.055452 | 1.03913; 1.07203 | <0.001 |
| 24-hour weighted rail, 60-65 dB | 1.059626 | 1.038557; 1.081122 | <0.001 |
| 24-hour weighted rail, 65-70 dB | 1.093471 | 1.058516; 1.12958 | <0.001 |
| 24-hour weighted rail, 70-75 dB | 1.099999 | 1.035328; 1.16871 | 0.002 |
| 24-hour weighted rail, 75+ dB | 0.959725 | 0.8290965; 1.110935 | 0.582 |
| **All causes of death, all persons, both sexes, per 5 dB, compared to lowest noise band** |  |  |  |
| Night-time road | 1.0186 | 1.013247; 1.023981 | <0.001 |
| Night-time rail | 1.005657 | 0.9959298; 1.01548 | 0.255 |
| 24-hour weighted road | 1.015397 | 1.009532; 1.021296 | <0.001 |
| 24-hour weighted rail | 1.007111 | 0.9941205; 1.020271 | 0.285 |

Annex table A2: Effect estimates (IRR) for all causes of death, all persons, both sexes.

|  | IRR | 95% confidence interval | p-value |
| --- | --- | --- | --- |
| **All causes of death, nationals, both sexes, per 5 dB, compared to no exposure** |  |  |  |
| Night-time road | 1.026654 | 1.023549; 1.029767 | <0.001 |
| Night-time rail | 1.027294 | 1.022313; 1.032299 | <0.001 |
| 24-hour weighted road | 1.028769 | 1.025429; 1.03212 | <0.001 |
| 24-hour weighted rail | 1.030848 | 1.024481; 1.037254 | <0.001 |
| **All causes of death, nationals, both sexes, each noise band compared to no exposure** |  |  |  |
| Night-time road, 45-50 dB | 1.038624 | 1.027896; 1.049464 | <0.001 |
| Night-time road, 50-55 dB | 1.069081 | 1.05417; 1.084203 | <0.001 |
| Night-time road, 55-60 dB | 1.069388 | 1.050888; 1.088214 | <0.001 |
| Night-time road, 60-65 dB | 1.120337 | 1.098579; 1.142527 | <0.001 |
| Night-time road, 65-70 dB | 1.101323 | 1.066267; 1.137532 | <0.001 |
| Night-time road, 70+ dB | 1.182287 | 1.022447; 1.367114 | 0.024 |
| Night-time rail, 45-50 dB | 1.054484 | 1.040192; 1.068972 | <0.001 |
| Night-time rail, 50-55 dB | 1.056897 | 1.038993; 1.07511 | <0.001 |
| Night-time rail, 55-60 dB | 1.079697 | 1.052349; 1.107756 | <0.001 |
| Night-time rail, 60-65 dB | 1.10597 | 1.059247; 1.154754 | <0.001 |
| Night-time rail, 65-70 dB | 1.017325 | 0.9262518; 1.117353 | 0.720 |
| Night-time rail, 70+ dB | 0.967965 | 0.7770742; 1.205747 | 0.771 |
| 24-hour weighted road, 55-60 dB | 1.052359 | 1.041423; 1.063411 | <0.001 |
| 24-hour weighted road, 60-65 dB | 1.062679 | 1.047447; 1.078133 | <0.001 |
| 24-hour weighted road, 65-70 dB | 1.079277 | 1.060199; 1.098697 | <0.001 |
| 24-hour weighted road, 70-75 dB | 1.12328 | 1.100418; 1.146618 | <0.001 |
| 24-hour weighted road, 75+ dB | 1.103743 | 1.057584; 1.151917 | <0.001 |
| 24-hour weighted rail, 55-60 dB | 1.05458 | 1.038037; 1.071386 | <0.001 |
| 24-hour weighted rail, 60-65 dB | 1.060993 | 1.039536; 1.082893 | <0.001 |
| 24-hour weighted rail, 65-70 dB | 1.093236 | 1.057542; 1.130134 | <0.001 |
| 24-hour weighted rail, 70-75 dB | 1.103728 | 1.037517; 1.174164 | 0.002 |
| 24-hour weighted rail, 75+ dB | 0.958058 | 0.8232902; 1.114886 | 0.580 |
| **All causes of death, nationals, both sexes, per 5 dB, compared to lowest noise band** |  |  |  |
| Night-time road | 1.019871 | 1.014388; 1.025384 | <0.001 |
| Night-time rail | 1.006719 | 0.9968077; 1.016729 | 0.185 |
| 24-hour weighted road | 1.017054 | 1.011044; 1.0231 | <0.001 |
| 24-hour weighted rail | 1.00881 | 0.9955392; 1.022258 | 0.194 |

Annex table A3: Effect estimates (IRR) for all causes of death, nationals, both sexes.

|  | IRR | 95% confidence interval | p-value |
| --- | --- | --- | --- |
| **Deaths from IHD, remaining persons, both sexes, per 5 dB, compared to no exposure** |  |  |  |
| Night-time road | 1.028588 | 1.020904; 1.036331 | <0.001 |
| Night-time rail | 1.03499 | 1.022656; 1.047473 | <0.001 |
| 24-hour weighted road | 1.031955 | 1.02369; 1.040288 | <0.001 |
| 24-hour weighted rail | 1.040459 | 1.024702; 1.056458 | <0.001 |
| **Deaths from IHD, remaining persons, both sexes, each noise band compared to no exposure** |  |  |  |
| Night-time road, 45-50 dB | 1.047437 | 1.0208; 1.07477 | <0.001 |
| Night-time road, 50-55 dB | 1.092696 | 1.055602; 1.131093 | <0.001 |
| Night-time road, 55-60 dB | 1.059124 | 1.014022; 1.106233 | 0.001 |
| Night-time road, 60-65 dB | 1.118807 | 1.065636; 1.174631 | <0.001 |
| Night-time road, 65-70 dB | 1.123387 | 1.037364; 1.216544 | 0.004 |
| Night-time road, 70+ dB | 1.138745 | 0.7877807; 1.646069 | 0.485 |
| Night-time rail, 45-50 dB | 1.057238 | 1.022036; 1.093652 | 0.001 |
| Night-time rail, 50-55 dB | 1.064623 | 1.020399; 1.110764 | 0.004 |
| Night-time rail, 55-60 dB | 1.099052 | 1.031461; 1.171072 | 0.004 |
| Night-time rail, 60-65 dB | 1.158061 | 1.042909; 1.285928 | 0.006 |
| Night-time rail, 65-70 dB | 1.121905 | 0.8978429; 1.401882 | 0.312 |
| Night-time rail, 70+ dB | 1.213256 | 0.7413753; 1.985487 | 0.442 |
| 24-hour weighted road, 55-60 dB | 1.071223 | 1.043926; 1.099234 | <0.001 |
| 24-hour weighted road, 60-65 dB | 1.088387 | 1.050453; 1.127691 | <0.001 |
| 24-hour weighted road, 65-70 dB | 1.066771 | 1.020295; 1.115364 | 0.004 |
| 24-hour weighted road, 70-75 dB | 1.129645 | 1.073541; 1.188681 | <0.001 |
| 24-hour weighted road, 75+ dB | 1.125646 | 1.013218; 1.250549 | 0.027 |
| 24-hour weighted rail, 55-60 dB | 1.065643 | 1.024765; 1.108153 | 0.001 |
| 24-hour weighted rail, 60-65 dB | 1.069398 | 1.016457; 1.125095 | 0.010 |
| 24-hour weighted rail, 65-70 dB | 1.120132 | 1.032248; 1.215498 | 0.007 |
| 24-hour weighted rail, 70-75 dB | 1.183213 | 1.019853; 1.372741 | 0.026 |
| 24-hour weighted rail, 75+ dB | 1.103717 | 0.7746061; 1.572659 | 0.585 |
| **Deaths from IHD, remaining persons, both sexes, per 5 dB, compared to lowest noise band** |  |  |  |
| Night-time road | 1.017151 | 1.003644; 1.03084 | 0.013 |
| Night-time rail | 1.021696 | 0.9971668; 1.046828 | 0.083 |
| 24-hour weighted road | 1.011627 | 0.9968815; 1.026591 | 0.123 |
| 24-hour weighted rail | 1.022768 | 0.9900469; 1.056571 | 0.175 |

Annex table A4: Effect estimates (IRR) for death from ischemic heart disease (IHD), remaining persons, both sexes.

|  | IRR | 95% confidence interval | p-value |
| --- | --- | --- | --- |
| **Deaths from IHD, all persons, both sexes, per 5 dB, compared to no exposure** |  |  |  |
| Night-time road | 1.026112 | 1.018446; 1.033835 | <0.001 |
| Night-time rail | 1.032586 | 1.020279; 1.045041 | <0.001 |
| 24-hour weighted road | 1.029255 | 1.021011; 1.037565 | <0.001 |
| 24-hour weighted rail | 1.037599 | 1.021883; 1.053557 | <0.001 |
| **Deaths from IHD, all persons, both sexes, each noise band compared to no exposure** |  |  |  |
| Night-time road, 45-50 dB | 1.042866 | 1.016345; 1.070079 | 0.001 |
| Night-time road, 50-55 dB | 1.088555 | 1.051603; 1.126807 | <0.001 |
| Night-time road, 55-60 dB | 1.052248 | 1.007438; 1.09905 | 0.022 |
| Night-time road, 60-65 dB | 1.109102 | 1.056393; 1.164442 | <0.001 |
| Night-time road, 65-70 dB | 1.107622 | 1.022806; 1.199472 | 0.012 |
| Night-time road, 70+ dB | 1.10647 | 0.7654527; 1.599415 | 0.590 |
| Night-time rail, 45-50 dB | 1.0542 | 1.0191; 1.09051 | 0.002 |
| Night-time rail, 50-55 dB | 1.059122 | 1.015126; 1.105025 | 0.008 |
| Night-time rail, 55-60 dB | 1.092434 | 1.02525; 1.164021 | 0.006 |
| Night-time rail, 60-65 dB | 1.148397 | 1.034206; 1.275197 | 0.010 |
| Night-time rail, 65-70 dB | 1.109893 | 0.88823; 1.386873 | 0.359 |
| Night-time rail, 70+ dB | 1.190105 | 0.7272281; 1.947601 | 0.489 |
| 24-hour weighted road, 55-60 dB | 1.067063 | 1.039873; 1.094965 | <0.001 |
| 24-hour weighted road, 60-65 dB | 1.083548 | 1.045783; 1.122677 | <0.001 |
| 24-hour weighted road, 65-70 dB | 1.058956 | 1.012821; 1.107193 | 0.012 |
| 24-hour weighted road, 70-75 dB | 1.118566 | 1.063012; 1.177024 | <0.001 |
| 24-hour weighted road, 75+ dB | 1.107229 | 0.9966398; 1.230089 | 0.058 |
| 24-hour weighted rail, 55-60 dB | 1.061429 | 1.020712; 1.10377 | 0.003 |
| 24-hour weighted rail, 60-65 dB | 1.064504 | 1.011806; 1.119946 | 0.016 |
| 24-hour weighted rail, 65-70 dB | 1.109657 | 1.022595; 1.204132 | 0.013 |
| 24-hour weighted rail, 70-75 dB | 1.174336 | 1.012201; 1.362441 | 0.034 |
| 24-hour weighted rail, 75+ dB | 1.086124 | 0.7622588; 1.547593 | 0.647 |
| **Deaths from IHD, all persons, both sexes, per 5 dB, compared to lowest noise band** |  |  |  |
| Night-time road | 1.015244 | 1.00177; 1.028899 | 0.026 |
| Night-time rail | 1.019798 | 0.9953168; 1.044882 | 0.114 |
| 24-hour weighted road | 1.009365 | 0.9946578; 1.024289 | 0.213 |
| 24-hour weighted rail | 1.020758 | 0.9881078; 1.054487 | 0.215 |

Annex table A5: Effect estimates (IRR) for death from ischemic heart disease (IHD), all persons, both sexes.

|  | IRR | 95% confidence interval | p-value |
| --- | --- | --- | --- |
| **Deaths from IHD, nationals, both sexes, per 5 dB, compared to no exposure** |  |  |  |
| Night-time road | 1.027358 | 1.019581; 1.035195 | <0.001 |
| Night-time rail | 1.034208 | 1.021754; 1.046814 | <0.001 |
| 24-hour weighted road | 1.030688 | 1.022321; 1.039124 | <0.001 |
| 24-hour weighted rail | 1.039385 | 1.023459; 1.05556 | <0.001 |
| **Deaths from IHD, nationals, both sexes, each noise band compared to no exposure** |  |  |  |
| Night-time road, 45-50 dB | 1.045939 | 1.019096; 1.073489 | 0.001 |
| Night-time road, 50-55 dB | 1.089898 | 1.052536; 1.128586 | <0.001 |
| Night-time road, 55-60 dB | 1.055457 | 1.010012; 1.102946 | 0.016 |
| Night-time road, 60-65 dB | 1.114124 | 1.060408; 1.170561 | <0.001 |
| Night-time road, 65-70 dB | 1.114331 | 1.027312; 1.208721 | <0.001 |
| Night-time road, 70+ dB | 1.13449 | 0.7788773; 1.652466 | 0.511 |
| Night-time rail, 45-50 dB | 1.053769 | 1.01837; 1.090397 | 0.003 |
| Night-time rail, 50-55 dB | 1.064399 | 1.019785; 1.110965 | 0.004 |
| Night-time rail, 55-60 dB | 1.096263 | 1.02812; 1.168923 | 0.005 |
| Night-time rail, 60-65 dB | 1.152529 | 1.036566; 1.281464 | 0.009 |
| Night-time rail, 65-70 dB | 1.139063 | 0.9109125; 1.424358 | 0.254 |
| Night-time rail, 70+ dB | 1.174695 | 0.7061913; 1.954015 | 0.535 |
| 24-hour weighted road, 55-60 dB | 1.069181 | 1.041679; 1.097409 | <0.001 |
| 24-hour weighted road, 60-65 dB | 1.085892 | 1.047653; 1.125527 | <0.001 |
| 24-hour weighted road, 65-70 dB | 1.061777 | 1.014956; 1.110757 | 0.009 |
| 24-hour weighted road, 70-75 dB | 1.124621 | 1.067894; 1.18436 | <0.001 |
| 24-hour weighted road, 75+ dB | 1.119424 | 1.005397; 1.246383 | 0.040 |
| 24-hour weighted rail, 55-60 dB | 1.063914 | 1.022749; 1.106736 | 0.002 |
| 24-hour weighted rail, 60-65 dB | 1.066229 | 1.012841; 1.122431 | 0.014 |
| 24-hour weighted rail, 65-70 dB | 1.116782 | 1.028094; 1.21312 | 0.009 |
| 24-hour weighted rail, 70-75 dB | 1.184243 | 1.019045; 1.37622 | 0.027 |
| 24-hour weighted rail, 75+ dB | 1.096992 | 0.7649503; 1.573163 | 0.615 |
| **Deaths from IHD, nationals, both sexes, per 5 dB, compared to lowest noise band** |  |  |  |
| Night-time road | 1.016055 | 1.002369; 1.029929 | 0.021 |
| Night-time rail | 1.022474 | 0.9976788; 1.047886 | 0.076 |
| 24-hour weighted road | 1.010736 | 0.9957888; 1.025908 | 0.160 |
| 24-hour weighted rail | 1.022696 | 0.9895965; 1.056902 | 0.181 |

Annex table A6: Effect estimates (IRR) for death from ischemic heart disease (IHD), nationals, both sexes.

| 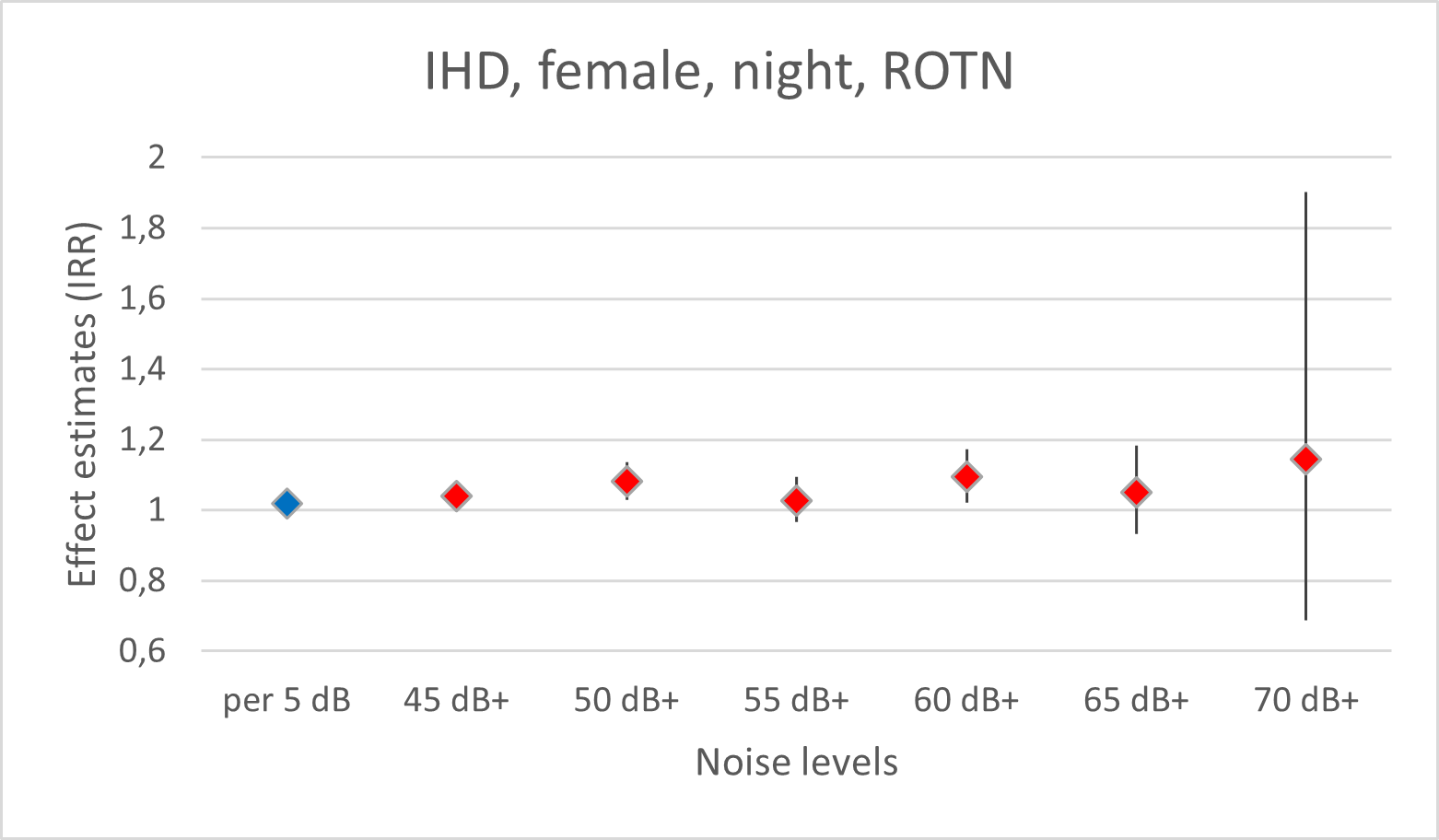 | 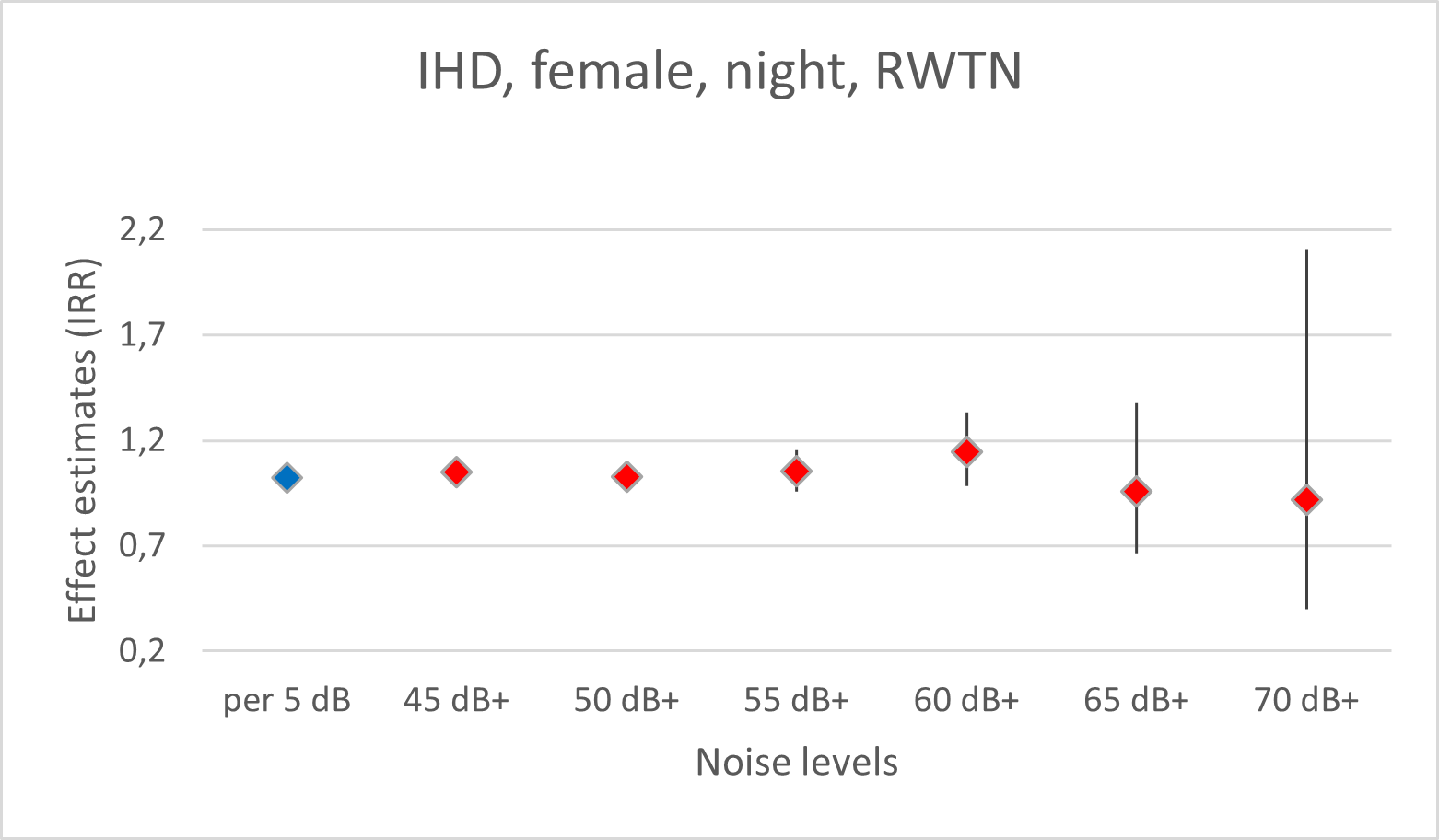 |
| --- | --- |
| 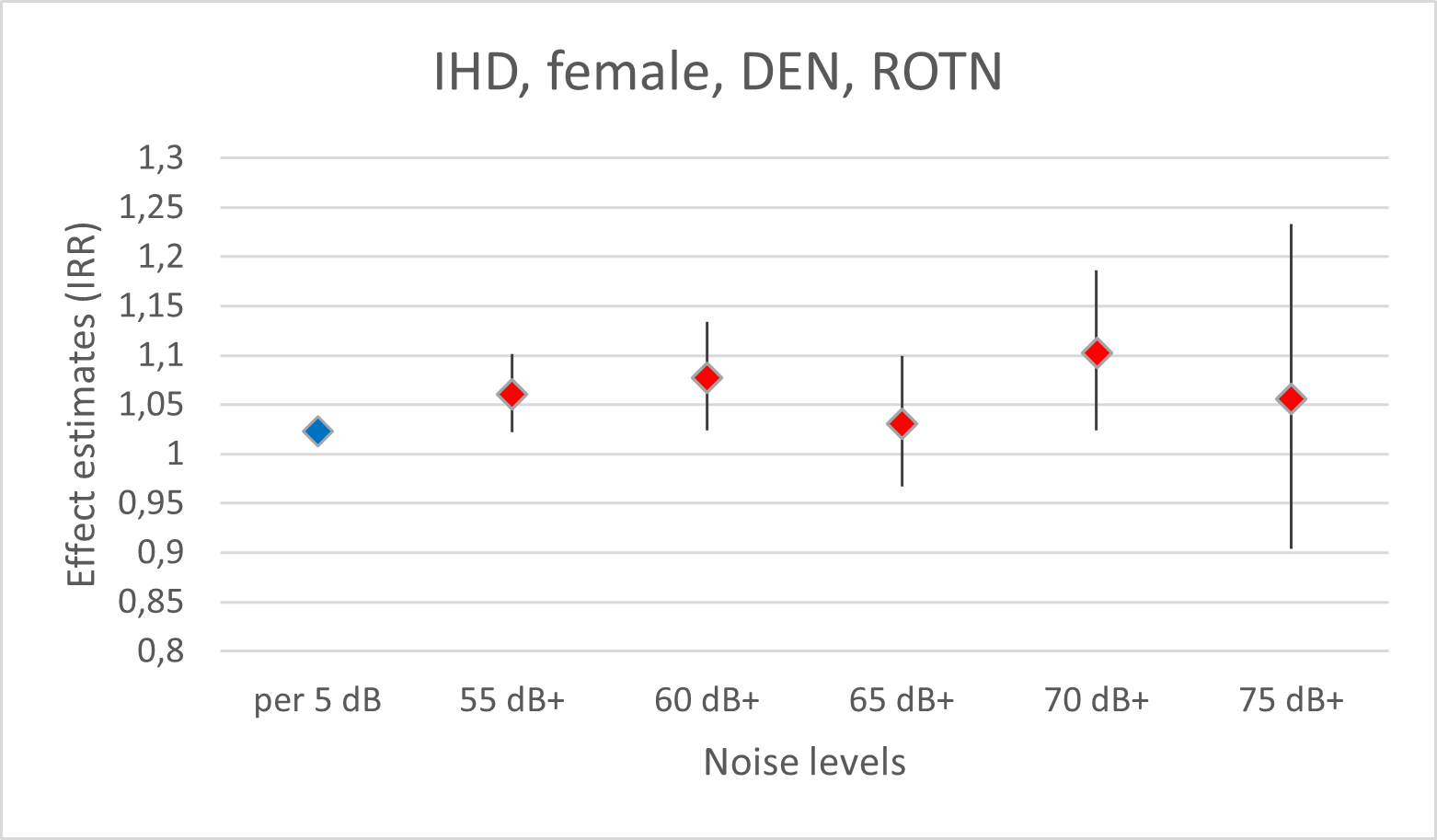 | 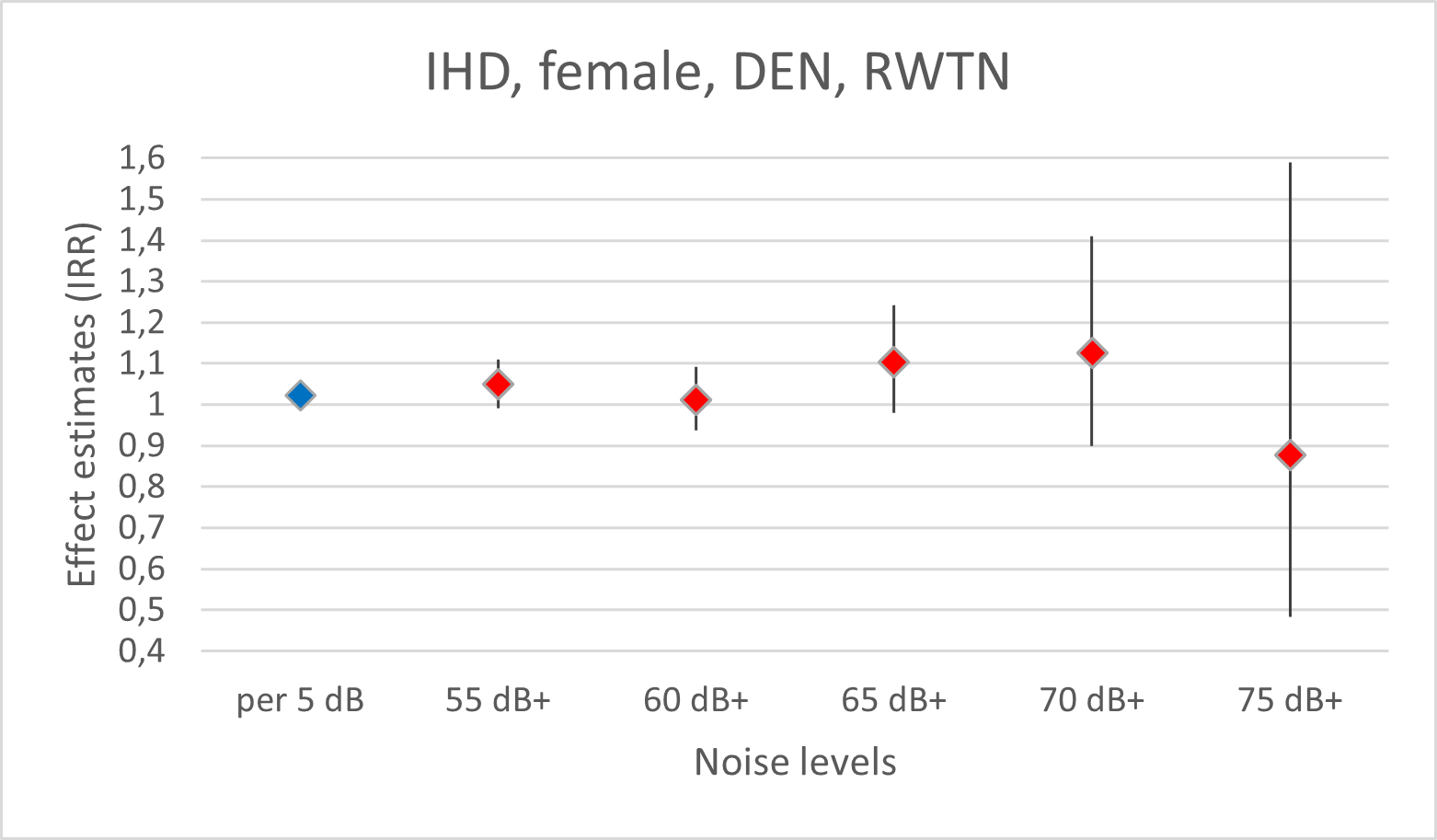 |

Annex figure A1: Effect estimates (IRR) per 5 dB increase in noise levels and/or compared to the non-exposed as the reference regarding deaths from ischemic heart disease, for females, at night (upper panel) and DEN-weighted 24-hour noise levels (lower panel) for ROTN (left) and RWTN (right). Data are based on “remaining persons”.

| 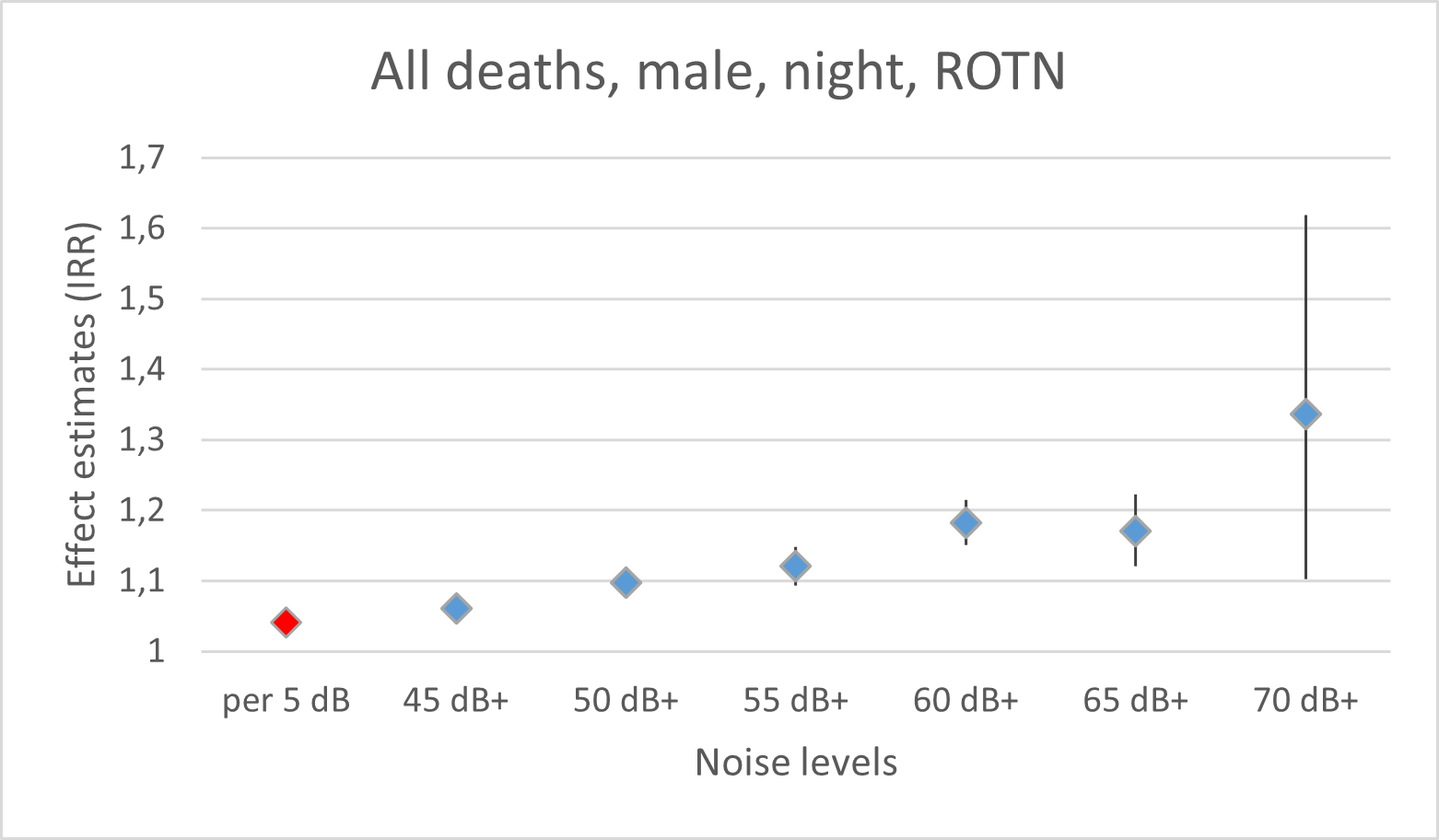 | 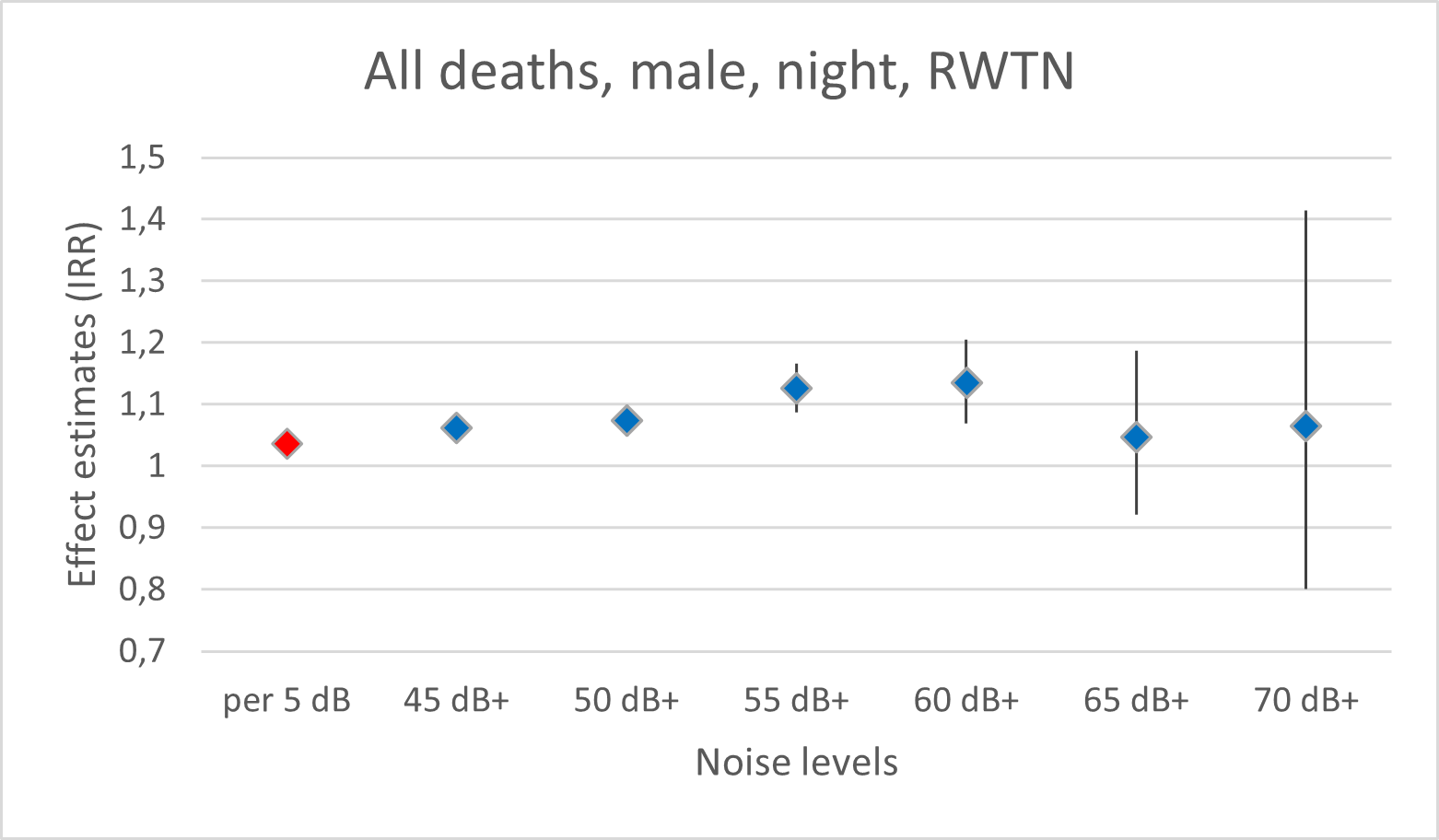 |
| --- | --- |
| 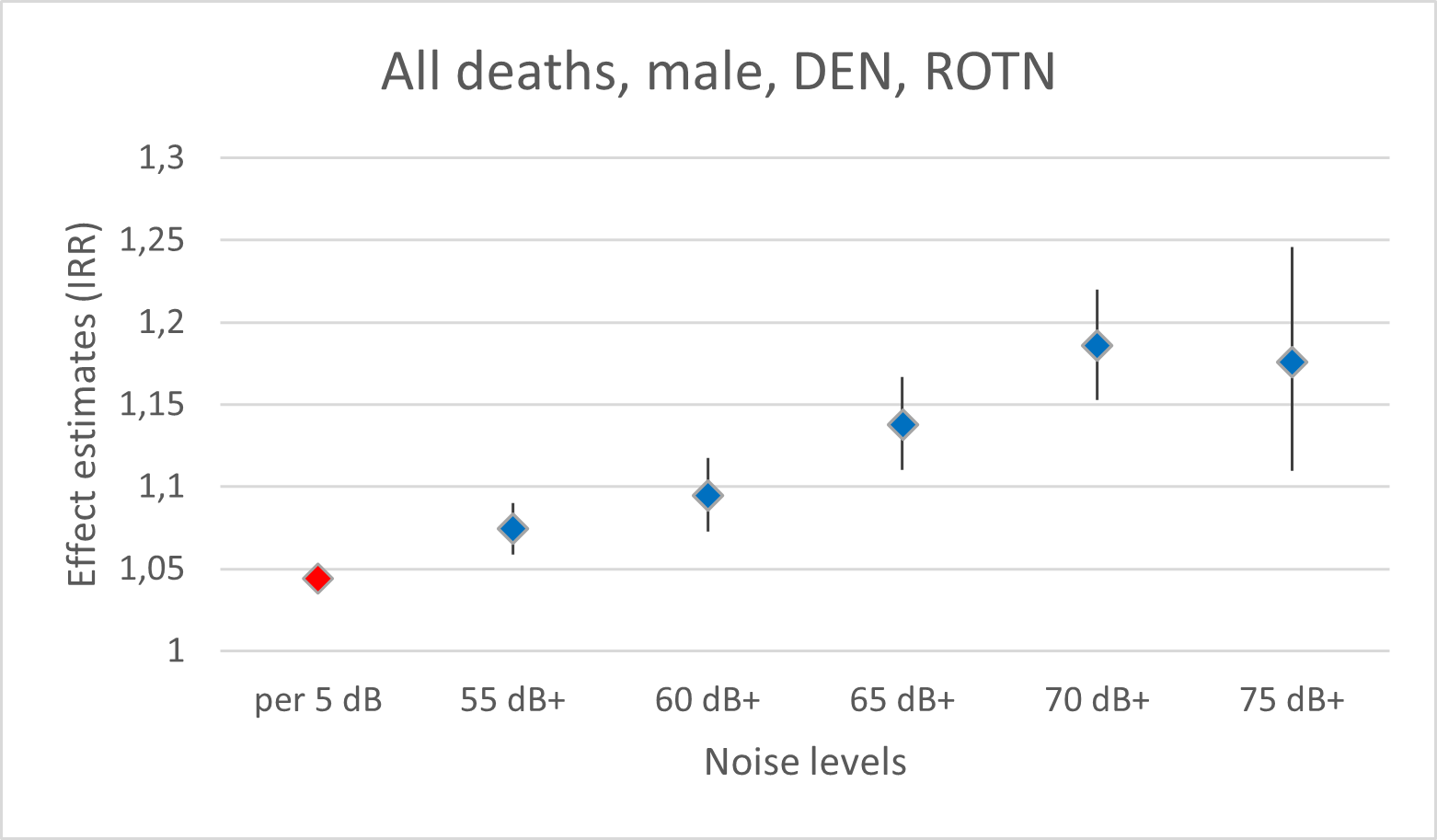 | 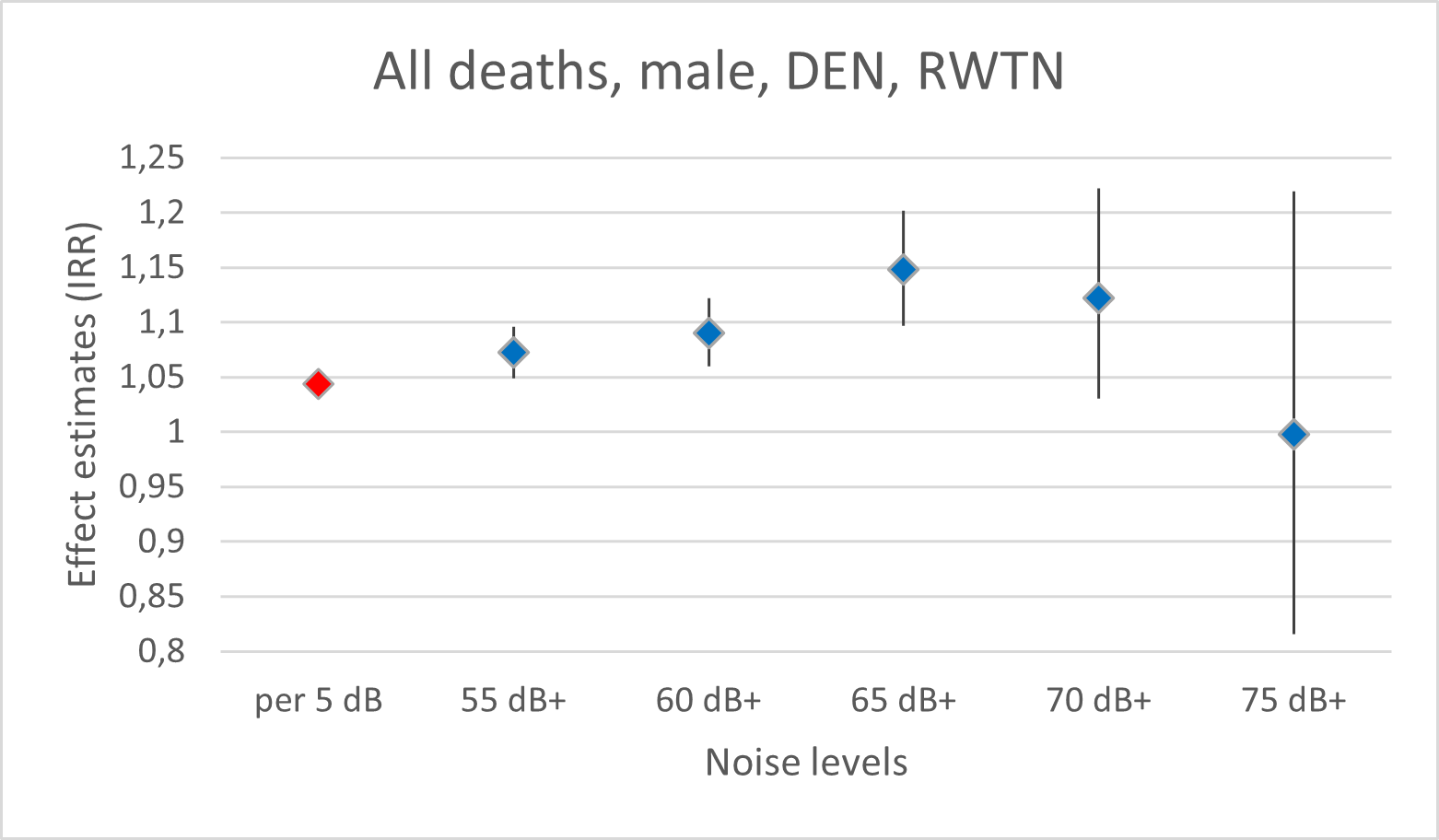 |

Annex figure A2: Effect estimates (IRR) per 5 dB increase in noise levels and/or compared to the non-exposed as the reference regarding deaths from all causes, for males, at night (upper panel) and DEN-weighted 24-hour noise levels (lower panel) for ROTN (left) and RWTN (right). Data are based on “remaining persons”.

| 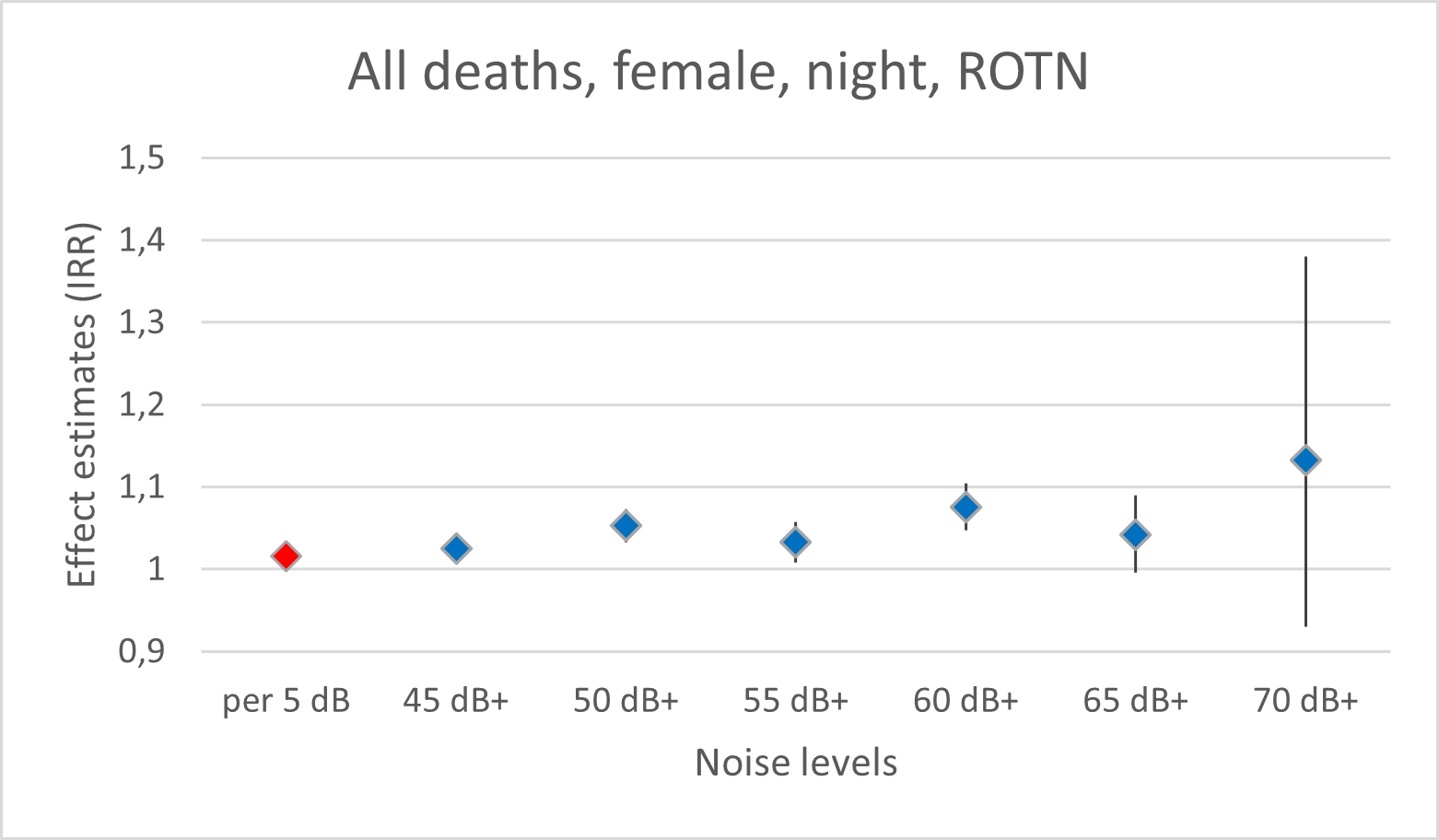 | 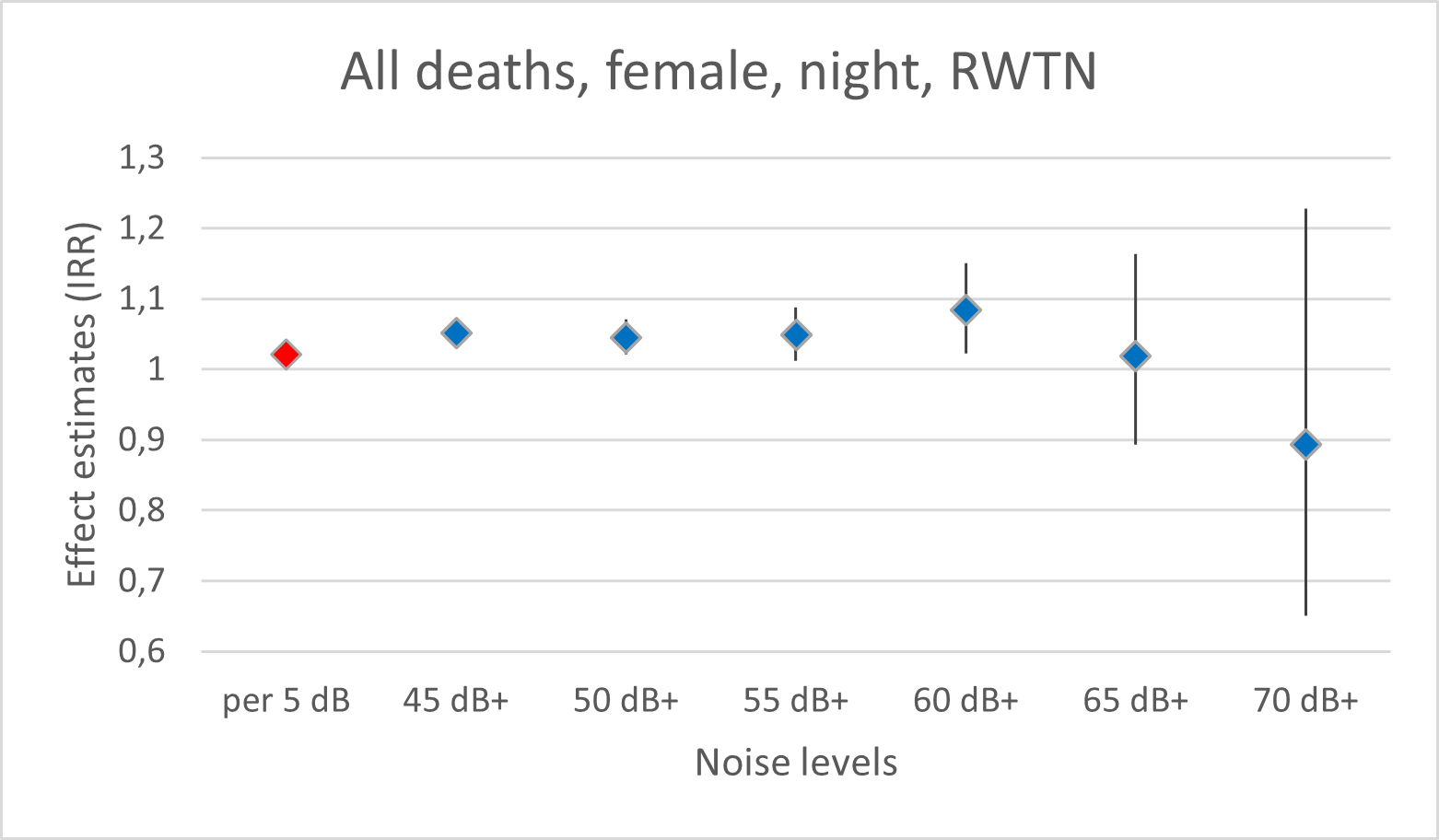 |
| --- | --- |
| 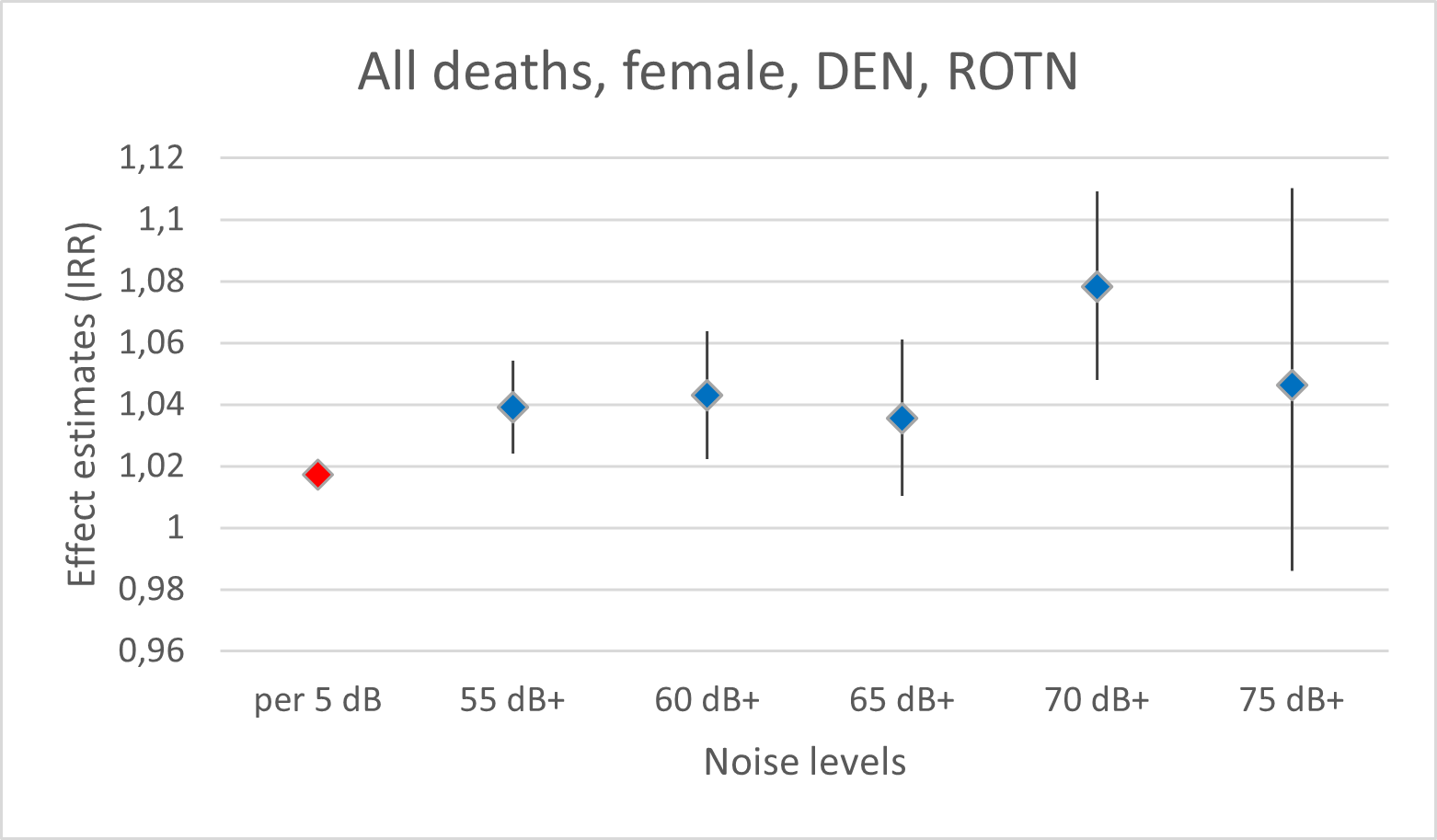 | 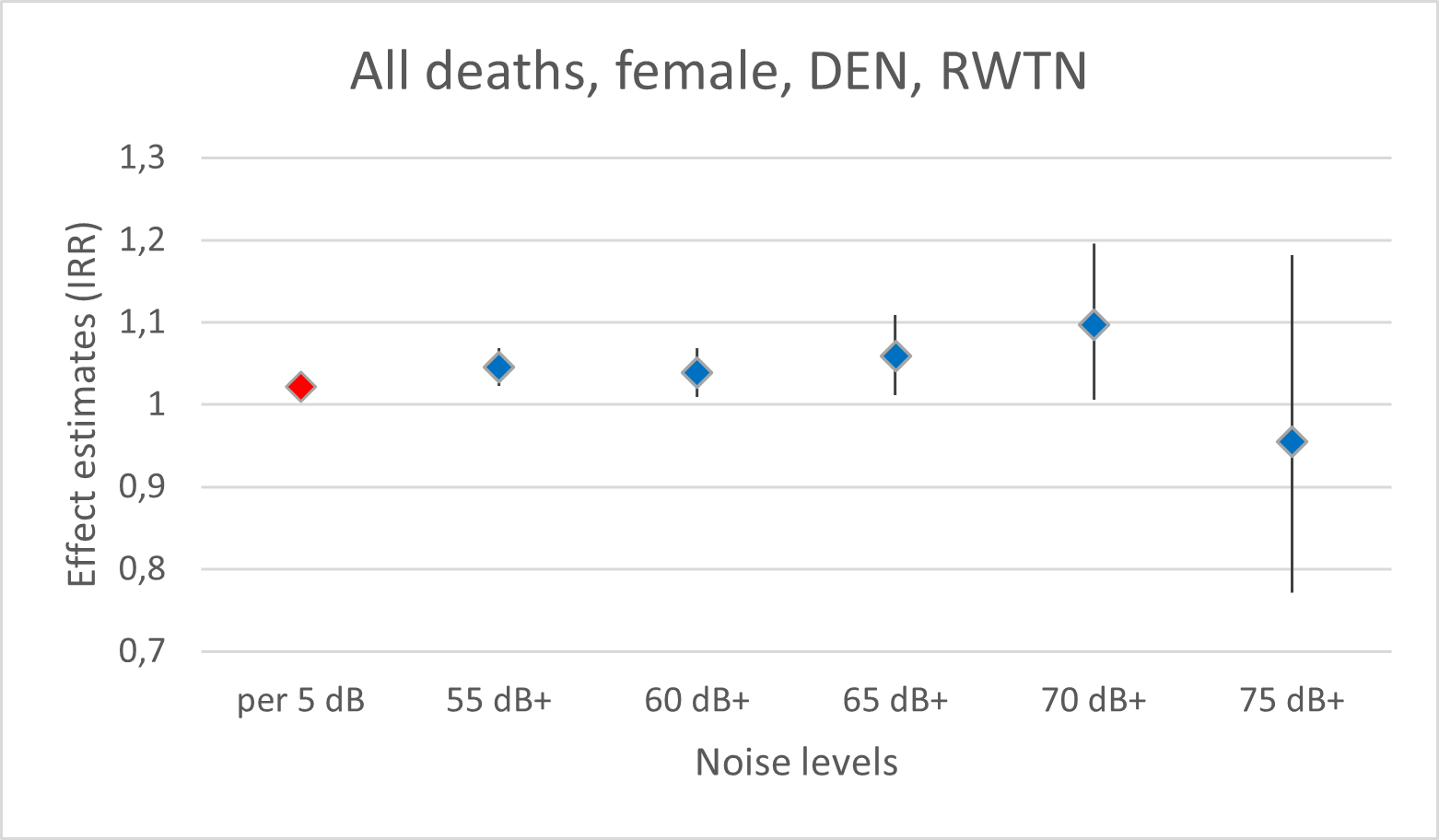 |

Annex figure A3: Effect estimates (IRR) per 5 dB increase in noise levels and/or compared to the non-exposed as the reference regarding deaths from all causes, for females, at night (upper panel) and DEN-weighted 24-hour noise levels (lower panel) for ROTN (left) and RTN (right). Data are based on “remaining persons”.


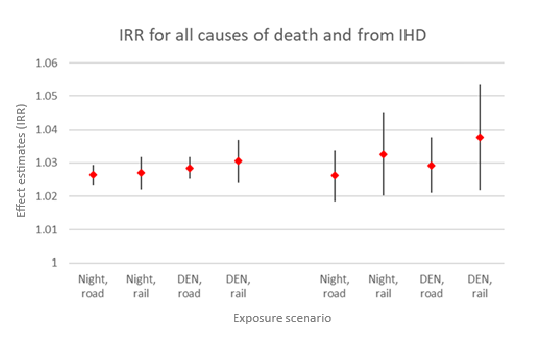


Annex figure A4: IRR calculated for both sexes combined, based on all persons, for all causes of death (left) and deaths from IHD (right).
